# Granularity mediated multiple reentrances with negative magnetoresistance in disordered TiN thin films

**DOI:** 10.1038/s41598-023-50091-7

**Published:** 2023-12-20

**Authors:** Sachin Yadav, R. P. Aloysius, Govind Gupta, Sangeeta Sahoo

**Affiliations:** 1 CSIR-National Physical Laboratory, Dr. K. S. Krishnan Marg, New Delhi, 110012 India; 2 Academy of Scientific and Innovative Research (AcSIR), Ghaziabad, 201002 India

**Keywords:** Physics, Condensed-matter physics, Superconducting properties and materials

## Abstract

Granular superconductors are the common examples of experimentally accessible model systems which can be used to explore various fascinating quantum phenomena that are fundamentally important and technologically relevant. One such phenomenon is the occurrence of reentrant resistive states in granular superconductors. Here, we report the observation of multiple reentrant resistive states for a disordered TiN thin film in its temperature and magnetic field dependent resistance measurements, *R*(*T*) and *R*(*B*), respectively. At each of the peak-temperatures corresponding to the zero-field *R*(*T*), a resistance peak appears in the *R*(*B*) around zero field which leads to a negative magnetoresistance (MR) region in its surrounding. These low-field negative MR regions appear for both perpendicular and parallel field directions with relatively higher amplitude and larger width for the parallel field. By adopting a granularity-based model, we show that the superconducting fluctuations in granular superconductors may lead to the observed reentrant states and the corresponding negative MR. Here, we propose that the reduction in the density of states in the fermionic channel due to the formation of Cooper pairs leads to the reentrant resistive state and the competition between the conduction processes in the single particle and Cooper channels result into the multiple resistive reentrances.

## Introduction

Granular superconductors offer suitable platforms to explore various intriguing quantum phenomena that are fundamentally important and are also relevant for nano technological applications^1^. These materials possess tuneable electronic properties that are mainly governed by the grain size along with the material properties of individual grains^2,3^ and the intergranular coupling between the neighbouring grains^4^. Further, as the granular materials are known to be disordered, the nature of disorder and the properties at the grain boundaries are very important in governing the conduction mechanism^4,5^. One of the signatures for granular superconductors is the appearance of resistance peak in the zero-field temperature dependent resistance [*R*(*T*)] measurements above the superconducting transition temperature (*T*_c_)^6^. The enhanced resistance can be associated to electron–electron interaction (EEI) in strong disorder regime^7–9^, or to weak localization (WL) originating from quantum interference in the weak disorder regime^7–10^. Furthermore, for a disordered system with relatively large grains and moderately strong intergranular coupling, the superconducting fluctuations (SFs) can also lead to a resistance maximum which is very sensitive to magnetic field with respect to its position and magnitude^1^. Among the SFs, there are three major contributions, namely, the Aslamazov–Larkin (AL)^11^ contribution, Maki–Thompson (MT)^12,13^ contribution and the Density of states (DOS) contribution^3^ that are significant above the *T*_c_^3,14–16^. In the normal phase Above *T*_c_, an enhancement of conductivity occurs due to shunting with a parallel conductive channel formed by the fluctuating Cooper pairs (FCPs). The related contribution, known as Aslamazov–Larkin (AL) contribution, is dominant near the *T*_*c*_^11^. The contribution from the coherent scattering of quasiparticles that are generated by the broken FCPs before losing their phase coherence is known as Maki–Thompson (MT) contribution^12,13^ which can be significant even far from the *T*_*c*_^17^. Moreover, the contributions arising from Aslamazov–Larkin (AL) and the Maki–Thompson (MT) corrections to the conductivity are positive. Whereas, the DOS correction related to the reduction in density of states in the single-particle spectrum due to the formation of FCPs leads to a negative contribution to the conductivity. Hence, the competition between DOS and the combined effect of AL and MT together can lead to a resistance maximum in the *R*(*T*), provided the DOS contribution dominates^6^.

Here, by using conventional granular superconductor, we aim to experimentally probe this regime where the aforementioned counteractive components of SFs may lead to the appearance of resistance maximum in the *R*(*T*) measurements. Further with the help of magnetotransport measurements, we intend to investigate the evolution of the resistance maximum with magnetic field and temperature to examine the role of the individual contributions within the SFs in more detail. However, it becomes important to verify and establish the most plausible reason behind the resistance maximum as there are other prominent mechanisms such as EEI and WL that may lead to the similar observations. In this regard, magnetoresistance (MR) measurement is one of the most common ways to distinguish the phenomena EEI and WL^1,7^. Hence, the present work is mainly focused to probe and monitor the resistance maximum appearing in *R*(*T*) for a granular superconductor in the regime where mostly the SFs dominate the transport. For the execution of the same, we have selected granular TiN thin film as the model system through which temperature and magnetic field dependent resistance measurements *R*(*T*, *B*) are carried out.

The resistance peak appearing above *T*_c_ in zero-field *R*(*T*) leads to a reentrant resistive state for a system which undergoes metal-superconductor transition at low temperature. Reentrant resistive states are experimentally observed in various systems and most of the times, a single reentrant state surrounding a single resistance peak is observed^4,18–23^. However, the appearances of single as well as double reentrances are also reported at temperature far below the *T*_c_^24–27^ and/or with the application of magnetic field^28,29^. In this article, we report the observation of multiple reentrant resistive states in the zero-field *R*(*T*) measurements for granular TiN thin film above the *T*_*c*_. Here, each of the reentrant resistive states is associated with a resistance peak that appears for the temperature range where the contributions from SFs to the conductivity correction are expected to be significant^30,31^. Under an external magnetic field, the resistance peaks get suppressed as observed in the field dependent *R*(*T*) which indicates the EEI might not be the origin behind the observed resistance peaks^30^. Further, magnetic field dependent resistance *R*(*B*) measurements, carried out in both perpendicular and parallel field directions, also feature multiple reentrances that evolve with temperature. Precisely, a resistance peak leading to negative MR appears around zero-field in the *R*(*B*) isotherms for each of the temperature points (*T*_peak_) that correspond to resistance peaks in the zero-field *R*(*T*).

Negative MR at the peak temperature has been reported for short aluminium wire within only few Oe for the case of a single reentrance^18^. However, the field extent for the negative MR region is much higher here. Further, for strongly disordered superconducting thin films at *T* ≪ *T*_*c*_ and at high field, electron–electron interaction mediated negative MR appears at the vicinity of the magnetic field induced superconductor insulator quantum transition (SIT)^32–34^. For example, the observation of resistance peak in *R*(*T*) and negative MR have been reported in strongly disordered superconducting thin films of *a*:InO^35^, TiN^28,36^, Pb^37^, Al-Ge^38^, NbTiN^39^ at very low temperature and high magnetic field. At *T* < *T*_*c*_, negative MR around zero field appeared also for amorphous InO nanowires^40^ and crystalline Mo_2_C flakes^41^. Most of these cases, the granularity is explained as the origin of the observed negative MR where the Josephson coupling strength between individual superconducting grains competes with other relevant energies related to Coulomb interaction and finite size effects that are significant at low temperature for relatively smaller grain size in strong disorder limit^38,42^.

However, the negative MR around zero-field here is observed for moderately large grains in the weak disorder regime at temperature above the *T*_*c*_ and at low field range. Generally, WL can be accounted for the observed negative MR where the amplitude and the width of the MR peak strongly depend on the temperature^43^. For WL, the amplitude increases by lowering temperature and the width gets broadened with increasing temperature^44^. Nevertheless, the correction to the resistance due to WL is significantly low and it is of the order of 0.001 to 0.01^43^. In contrary to the WL scenario, here for any particular field orientation, the widths of these negative MR peaks remain the same for all the peak temperatures, whereas, their amplitude decreases with decreasing temperature. This indicates the influence of SFs in the appearance of negative magnetoresistance near zero-field rather than the influence of WL. Moreover, the amplitude and the width for the MR peaks appear to be stronger in the parallel field than that for the perpendicular direction which again rules out the possibility of WL being the cause of the observed reentrance and the corresponding negative MR^43^. Therefore, the most plausible origin of the observed reentrant states can be the SFs where the competition between the negative contribution from the DOS correction and the positive contribution from AL and MT corrections to the conductivity may lead to the development of the observed resistance peaks in the zero-field *R*(*T*). Furthermore, by using a granularity-based model, we have illustrated that the observed multiple resistive reentrant states can be originated by the SFs^6,42^.

## Results

**Figure 1:**
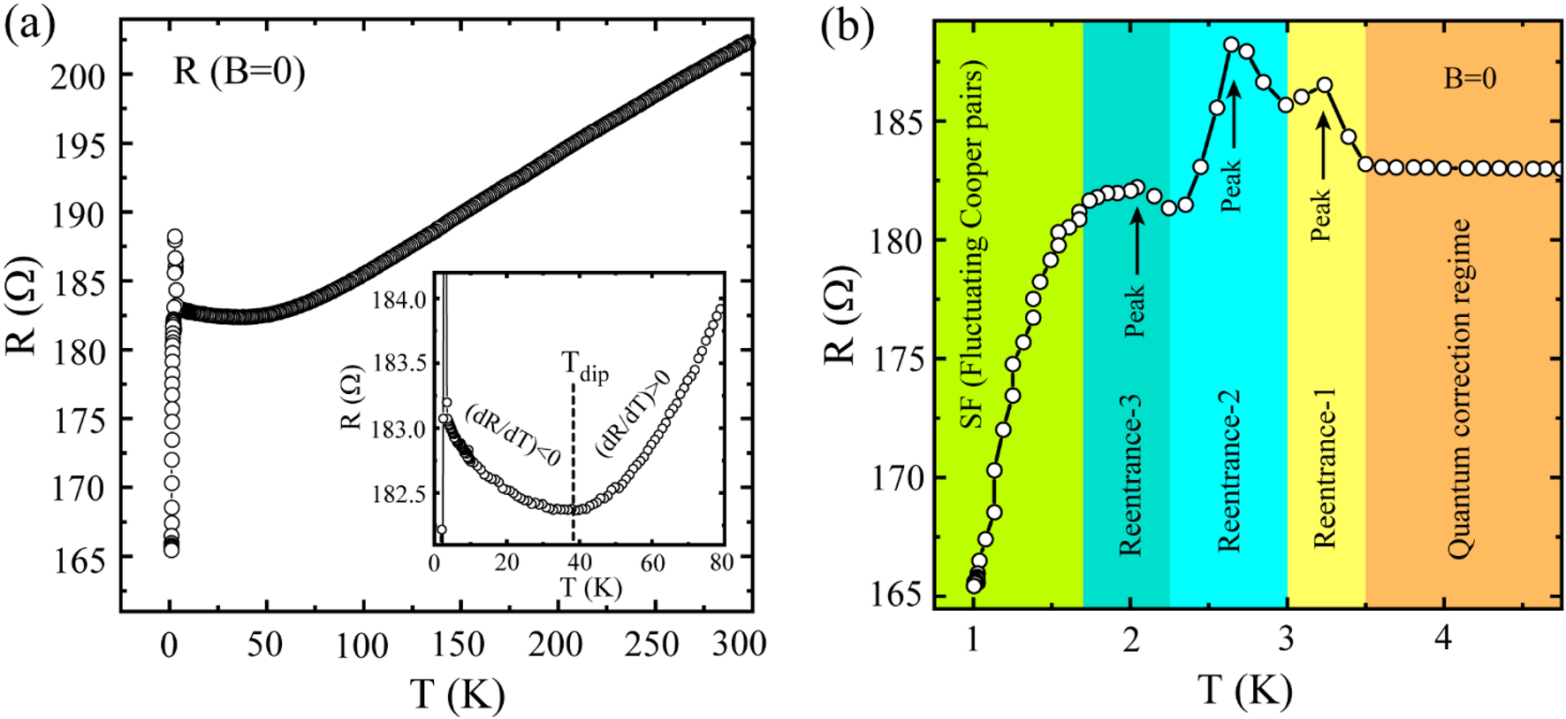
Temperature dependent resistance  *R*(*T*) measurements. (**a**) Full-scale  *R*(*T*) measurements under zero-field cooling down from room temperature to 1 K. Inset: A selected portion of the main panel is highlighted to show a resistance minimum at particular temperature (*T*_dip_) which eventually separates the negative and the positive d*R*/d*T* regions in the zero-field  *R*(*T*). (**b**) Different reentrance regions associated with the resistive peaks along with the superconducting fluctuations (mainly AL and MT contributions) regime and quantum corrections regime have been marked and highlighted in the low temperature zero-field  *R*(*T*).

First, we present the zero-field *R*(*T*) measurements of sample SS2 in Fig. 1. The full-scale *R*(*T*) measured from room temperature down to 1 K is presented in Fig. 1a which shows a metallic behaviour with positive d*R*/d*T* at high temperature and an upturn with negative d*R*/d*T* accompanying a resistive peak at low temperature. The crossover from the positive to negative d*R*/d*T* occurs at the characteristic temperature *T*_dip_ ~ 38 K which is shown by the dashed vertical line in the inset of Fig. 1a. For *T* < *T*_dip_, the resistance increases smoothly with decreasing temperature before a sudden upward jump in the resistance occurs which leads to resistance peak and the associated re-entrance by further lowering temperature.

We highlight the peak region and its immediate surroundings in Fig. 1b which defines three reentrant regions based on the observed three resistance peaks before transiting to the superconducting (SC) state. The peak positions are shown by the black upward arrows and the slope *dR/dT* changes its direction/sign at each of the peak temperature (*T*_peak_) points. The steep positive slope at temperatures below the Reentrance-3 region indicates the dominating role of the fluctuating Cooper pairs and hence the region is named as SF. The region between *T*_dip_ and the Reentrance-1 corresponds to the quantum corrections to the conductivity (QCC) regime. Further, *R*(*T*) measurements carried out under perpendicular magnetic field are shown in Fig. S1 in the Supporting Information (SI), where the resistive peaks get suppressed under the application of the field. This indicates towards WL and/or SFs as the possible mechanism(s) behind the upturn accompanying the resistive peaks.

**Figure 2:**
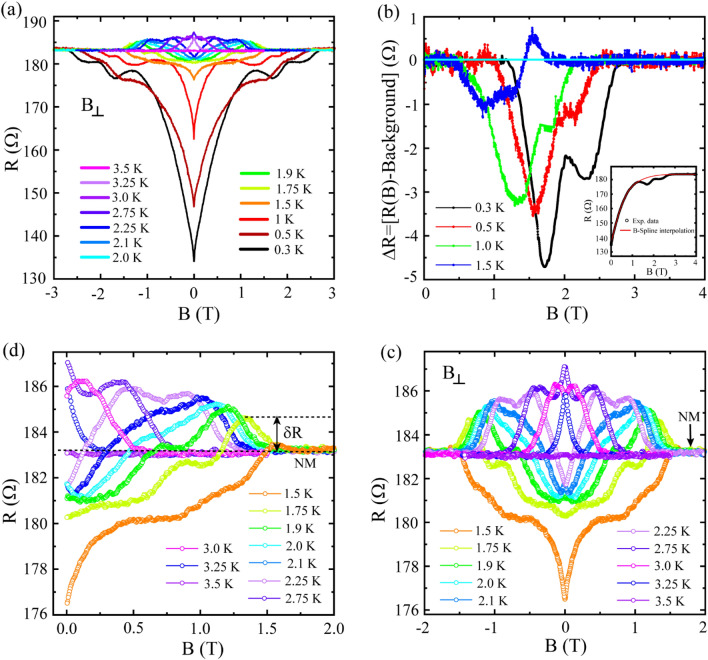
Magnetotransport measurements. (**a**) Magnetic field dependent resistance  *R*(*B*) isotherms measured under perpendicular magnetic field. The  *R*(*B*) isotherms measured in the temperature range from 300 mK up to the Reentrance-1 zone which ends at 3.5 K. (**b**) Δ*R*, representing the nonmonotonic oscillatory part embedded in the  *R*(*B*), obtained by subtracting a B-Spline interpolated background from the experimental  *R*(*B*) isotherms. Inset: The interpolated background (the red curve) along with the experimental data (the black curve) for a representative  *R*(*B*) isotherm measured at 300 mK. The details about the interpolated background are explained in the main text. The horizontal cyan line at Δ*R* = 0 indicates the overlapping of the background with the measured  *R*(*B*). (**c**) A selective set of  *R*(*B*) isotherms measured in the temperature window spanning over the reentrance regions. (d) The same set of  *R*(*B*) as presented in (c) but with only positive side of the *B*-axis for clarity.

Next, in Fig. 2, we present isothermal *R*(*B*) measurements carried out under perpendicular magnetic field. A set of *R*(*B*) isotherms measured at temperature between 300 mK and 3.5 K is displayed in Fig. 2a. From 300 mK up to 1.5 K, initially the resistance changes with a steep continuous positive slope up to a certain characteristic field above which the *R*(*B*) exhibits a non-trivial oscillatory/wavy type feature before merging onto the normal state (NM). For the *R*(*B*) isotherm measured at 300 mK, the characteristic field is ~ 1 T, above which the resistance follows a wavy path with a couple of grooves before reaching to the NM. For an insight into the oscillatory part, in Fig. 2b, we have subtracted the measured *R*(*B*) from an interpolated background which would likely be followed by the *R*(*B*) in absence of the observed bouncy features. For a representative *R*(*B*) data measured at 300 mK, the red solid curve in the inset of Fig. 2b presents the background which indeed follows the *R*(*B*) data except for the wavy part. This extra feature *ΔR*, as obtained after background subtraction, is shown in Fig. 2b for the temperature from 300 mK up to 1.5 K. The horizontal cyan line refers to the reference background with *ΔR* = *0*. At 300 mK, the non-zero *ΔR* appears only below this reference line for 1.1 T ≤ *B* ≤ 2.7 T and its maximum value is about ~ 4.8 Ω. With increasing temperature, the maximum amplitude of *ΔR* gets reduced and the feature moves towards lower field with reduced extent in field. At 1.5 K (the blue curve in Fig. 2b), some part goes above the background line before merging onto the NM. Further at higher temperature, the overall change in resistance is small and the extra features overlap with each other in the temperature evolution of the *R*(*B*) and for clarity, they have been presented separately in Fig. 2c,d.

A set of *R*(*B*) isotherms for *T* ≥ 1.5 K and for -2 T ≤ *B* ≤ 2 T is shown in Fig. 2c and for a clearer view, only the positive field side is presented in Fig. 2d. The wavy part, gets more prominent with increasing temperature and the segment appearing above the normal state grows in resistance. For example, at 2.25 K, a very tiny region of *R*(*B*) close to zero-field remains underneath the NM and the majority of the *R*(*B*) goes above it with a combination of positive and negative magnetoresistance regions before merging onto the NM. And for *T* ≥ 2.75 K, the nonmonotonic feature in *R*(*B*) stays fully above the NM and with increasing temperature, it moves towards the zero-field. For a quantitative analysis, we define the difference between the *R*_*N*_ and the maximum resistance appearing above the NM as *δR* (shown in Fig. 2d for the representative *R*(*B*) measured at 1.75 K). With increasing temperature, *δR* increases and attains the maximum value at 2.75 K which is exactly the same temperature where the zero-field *R*(*T*) shows the maximum resistance as shown in Fig. 1.

**Figure 3:**
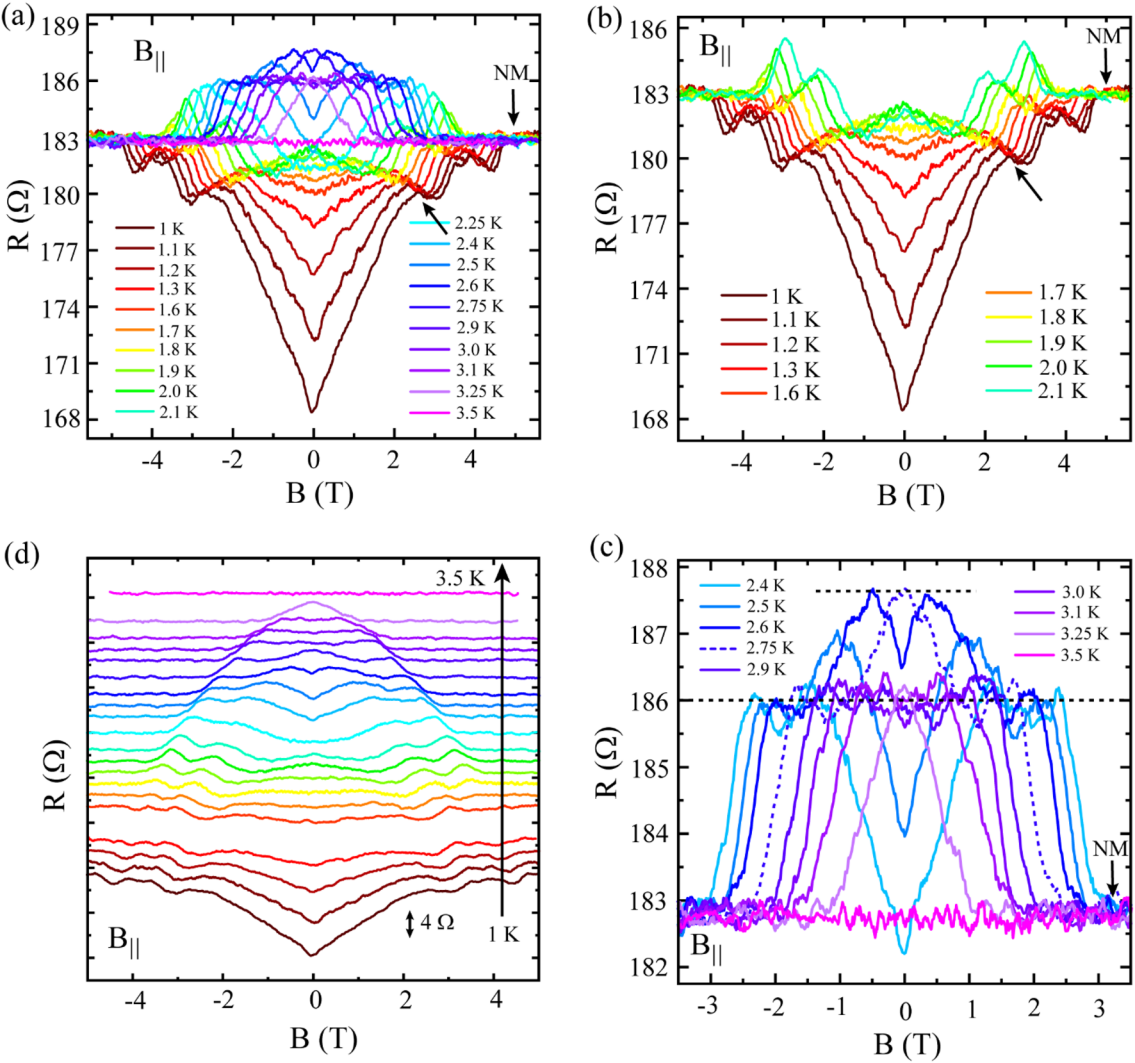
Magnetoresistance measurements under parallel magnetic field. (**a**)  *R*(*B*) isotherms measured in the temperature window of 1 K to 3.5 K. Similar to perpendicular field,  *R*(*B*) characteristics under parallel field also showcase oscillatory pattern containing multiple kink type of features that evolve with temperature. The horizontal curve related to R(B) measured at 3.5 K represents the normal state. The two parts below and above the normal state from (**a**) have been presented in (**b**) and (**c**), respectively. (**d**) The same set of  *R*(*B*) isotherms shifted linearly with temperature in the upward direction for clarity. The bottom curve measured at 1 K represents the original device resistance.

In Fig. 3, a set of *R*(*B*) isotherms measured under the field applied parallel to the current direction is shown in different representations. The *R*(*B*) isotherms for 1 K ≤ *T* ≤ 3.5 K are shown in Fig. 3a where similar type of wavy features are observed as that appeared for the perpendicular field. Eventually, with increasing temperature, the overall *R*(*B*) curves move towards the NM at around 183 Ω as represented by the horizontal magenta curve measured at 3.5 K. While increasing temperature from 1.0 to 2.0 K, the first kink (shown by the arrow in Fig. 3a) moves towards lower field and eventually, leads to a resistance peak (negative MR) around zero-field which is just opposite to the observed dip (positive MR) for the isotherm measured at 1 K. Hence, crossover from positive to negative MR around zero-field is observed with increasing temperature. With further increasing temperature, the rest of the kinks evolve with temperature in a similar fashion and the *R*(*B*) isotherms start to appear above the NM. For a detailed insight, this whole set of *R*(*B*) isotherms is split into two subsets; one appears below and the other one is above the normal state as shown in Fig. 3b,c, respectively. The negative MR peak around zero-field at 2.0 K is clear from Fig. 3b. It should be noted that the third peak at the centre of the Reentrance-3 regime in the zero-field *R*(*T*) (Fig. 1b) appears also at 2.0 K.

The top part from Fig. 3a is displayed in Fig. 3c where the temperature evolution of the *R*(*B*) isotherms features two common resistive steps with the resistance values of ~ 187.70 Ω and ~ 186 Ω as shown by the horizontal dashed black lines above the NM. Interestingly, these two specific resistance values closely match with the resistance values for the 2nd peak from the Reentrance-2 and the 1st peak from the Reentrance-1 region in the zero-field *R*(*T*)  Fig. 1b. Besides, the *R*(*B*) isotherms for 2.4 K ≤ *T* ≤ 3.0 K in Fig. 3c feature both positive and negative MR segments that evolve with the temperature. At 3.25 K, only a resistance peak around zero-field appears which indicates the presence of negative MR only. Finally, at 3.5 K, no traceable MR is observed and the *R*(*B*) isotherm shown by the magenta horizontal curve overlaps with the NM. For clarity, in Fig. 3d, the *R*(*B*) isotherms altogether is shifted linearly with temperature in the upward direction. Evidently, with increasing temperature, the wavy features in the *R*(*B*) isotherms move systematically towards lower field and finally the features converge at zero field for 3.25 K. Further, the temperature evolution of individual features is presented in detail in Fig. S2 in the SI.

**Figure 4:**
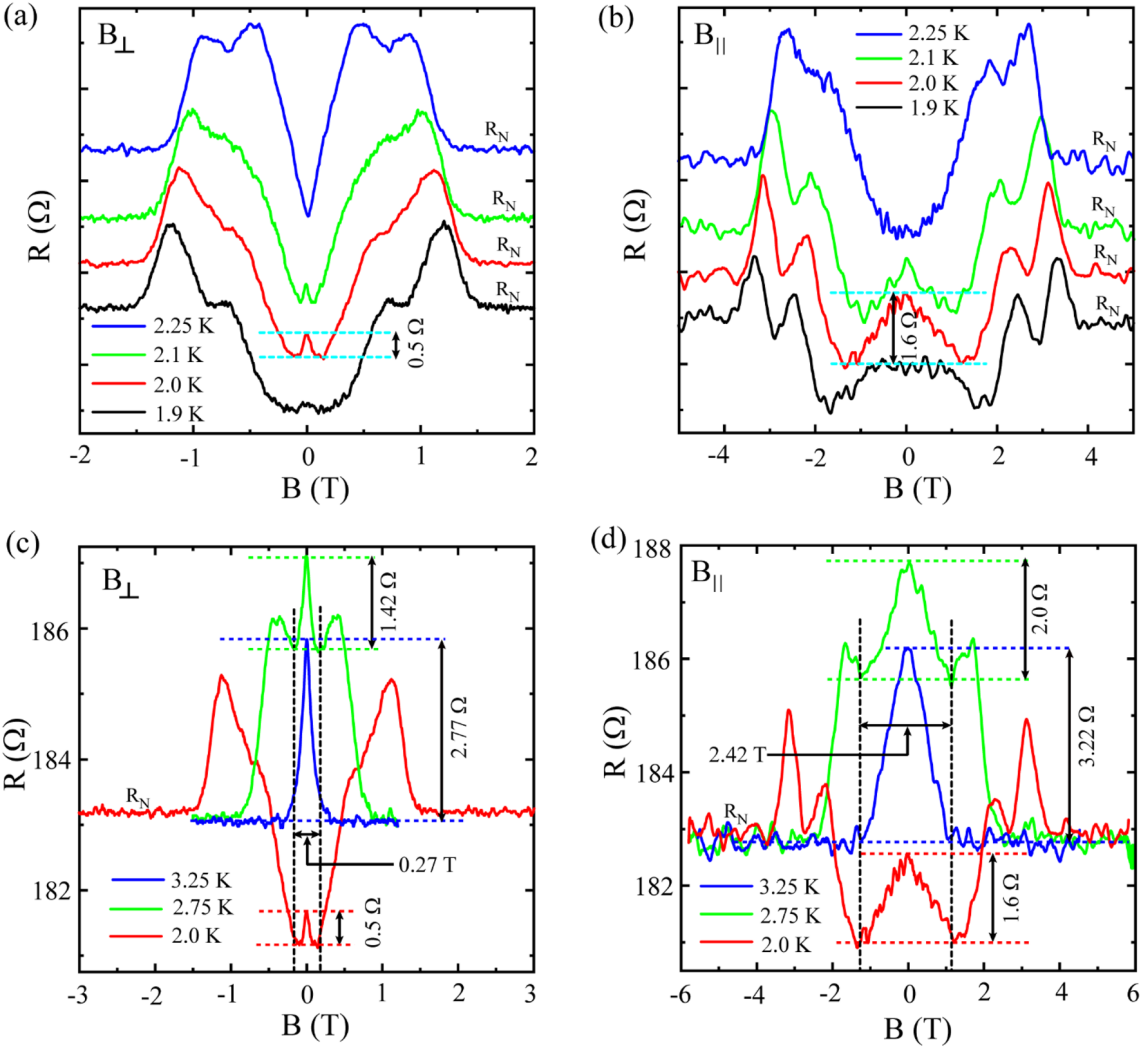
Comparison of  *R*(*B*) isotherms for parallel and perpendicular magnetic field orientations. Selective sets of isotherms for perpendicular field (**a**,**c**) and for parallel field (**b**,**d**). (**a**,**b**)  *R*(*B*) isotherms from Reentrance-3 region shifted linearly with temperature in the upward direction for clarity and the bottom black curve measured at 1.9 K represents the original sample resistance. (**c**,**d**) Isotherms displaying negative magnetoresistance by accompanying a resistance peak around zero-field. The corresponding temperature values match with the peak positions in the zero-field  *R*(*T*) data. Further, the peak resistance at *B* = 0, as obtained from the  *R*(*B*) isotherms, follows the same trend as it appeared in the zero-field  *R*(*T*). For example, the maximum resistance occurs for the peak at 2.75 K which matches exactly with the value that appeared in the zero-field  *R*(*T*) at the same temperature.

Next in Fig. 4, we compare the MR measurements for perpendicular and parallel field orientations. In this regard, *R*(*B*) isotherms for the temperature range covering the Reentrance-3 regime (Fig. 1b) are considered in Fig. 4a,b for perpendicular and parallel field orientations, respectively. The peak around zero-field starts to appear at 1.9 K. With increasing temperature, the peak gets prominent and finally, it converts into a dip at 2.25 K for both the field orientations. However, the amplitude and the width of the peak differ for perpendicular and parallel fields. One-to-one comparison of *R*(*B*) isotherms measured at the same temperature for parallel and perpendicular field directions is presented in Fig. S3 in the SI. More specifically, at each *T*_peak_, the peak around zero-field appears with higher amplitude and larger width for parallel field than that for perpendicular field. Here, we compare three specific *R*(*B*) isotherms measured at three peak temperatures 3.25 K, 2.75 K and 2.0 K, in Fig. 4c,d for perpendicular and parallel field orientations, respectively. The number of resistance peaks changes from 3 → 2 → 1 for changing the temperature from 2.0 K → 2.75 K → 3.25 K for both the field orientations. Similar to the peak at 2.0 K (Fig. 4a,b), the amplitude of the peak around zero-field for 2.75 K or 3.25 K is more prominent for parallel field than that for the perpendicular orientation. Interestingly, for any particular field direction, all the three zero-field MR peaks measured at these three specific peak temperatures are almost of the same width as indicated by the black vertical dotted lines in Fig. 4c,d. Generally, negative MR originated due to WL strongly depends on temperature and with increasing temperature, the peak width broadens up while the peak amplitude decreases. Therefore, WL alone might not be the origin of the negative MR here.

Nevertheless, we have calculated *MR* (*%*) [$MR\,(\%)=\frac{R\left(B\right)-R(0)}{R(0)}\times 100$, where *R*(0) is zero-field resistance] and for a set of representative isotherms measured under perpendicular field the *MR *(*%*) is shown in Fig. S4 in the SI, which defines the positions of various resistance peaks, normal state and the maximum MR state. In the studied range of temperature, *MR *(*%*) shows an oscillatory type of behaviour with decaying amplitude for higher temperature (Fig. S4b,c). The corresponding field-temperature dependence for all the characteristic states are shown in Fig. S5 in the SI for both the field orientations. Further, we observe that the excess resistance ‘*δR*’ (defined as the difference in the maximum resistance above the NM and the *R*_N_ as shown in Fig. 2d) varies with temperature in a similar fashion as that of the zero-field *R*(*T*) and the comparison is presented in Fig. S4d. The appearance of peaks in temperature dependent ‘*δR*’ at the same temperature as that appeared in zero-field *R*(*T*) and also the similar pattern of amplitude modulation of ‘*δR*’ and zero-field *R*(*T*) indicate that the same mechanism is responsible behind the appearance of peak structures in both *R*(*T*) and *R*(*B*).

## Discussion

The zero-field *R*(*T*) measurements in a disordered TiN thin film above the *T*_c_ exhibit multiple resistive reentrant states and the associated resistance peaks get suppressed under perpendicular magnetic field. This implies a negligible contribution from the EEI towards the observed resistance up-turn and the associated re-entrance. Further, the temperature evolution of the low-field MR peaks with respect to their amplitude, width and the field orientation indicates that WL cannot be accounted fully for the observed reentrant states^43,45^. As the sample appears to be granular in nature (Fig. S8), SFs might be the most plausible mechanism responsible for the observed reentrant states and the associated negative MR^37,46,47^. In this scenario, the Cooper pairs are localized in individual grains and the conduction is mainly led by the single particle conduction channel.

The manifestation of SFs in the normal phase of a superconductor occurs directly by opening a parallel shunting channel due to the formation of fluctuating Cooper pairs (FCPs) above the *T*_c_ which is known as the AL contribution^11^. On approaching the *T*_c_, it can be expressed in terms of the reduced temperature $\epsilon \equiv ln\left(\frac{T}{{T}_{c}}\right)\approx \frac{T-{T}_{c}}{{T}_{c}}\ll$ 1 in 2D with thickness *d* as^3^,

1 $${\sigma }^{AL}\left(T\right)\sim \frac{{e}^{2}}{\hslash d}\frac{1}{\epsilon }$$

Besides, there are two major indirect contributions of the SFs on the quasiparticle subsystem, namely, the MT and the DOS contributions that are significant above the *T*_c_. The former is relevant in the dirty limit and close to the *T*_c_ where the coherent scattering of FCPs with impurities leads to an increase in the conductivity in the normal phase of the single particle channel. In 2D, the MT contribution can be expressed as^3^,

2 $${\sigma }^{MT}\left(T\right)\sim \frac{{e}^{2}}{8\hslash }\frac{1}{\epsilon }ln\frac{{\tau }_{\varphi }}{{\tau }_{GL}}$$

With the Ginzburg–Landau relaxation time ${\tau }_{GL}=\frac{\pi \hslash }{8{k}_{B}\left(T-{T}_{c}\right)}$. Here, the dephasing time ${\tau }_{\phi }=\frac{\pi \hslash }{8{k}_{B}T}\frac{1}{\delta }$ introduces phase breaking processes due to mainly inelastic scattering with the pair breaking parameter δ^17,48^.

The DOS contribution originating from the depletion of quasiparticles for the formation of FCPs is given by^3^,

3 $${\sigma }^{DOS}\left(T\right)\sim -\frac{{e}^{2}}{\hslash }ln\frac{1}{\epsilon }$$

From Eqs. (1)–(3), the AL and MT contributions are positive, whereas, there is a negative sign in front of the DOS contribution. Here, the suppression in the DOS for the single particle channel results into increased resistance^23,49,50^. Further on approaching the *T*_c_, the AL and MT terms are singular in temperature whereas, the DOS shows relatively weaker dependence on temperature as evident by the slow varying logarithmic dependence of the temperature. At *T* close to *T*_c_, AL and MT dominate. MT contributions can be significant at temperature far from the *T*_c_ also, however, as it is very sensitive to the phase breaking processes, it can be suppressed by the application of magnetic field^6,17^. A little far from the *T*_c_, DOS can be significant and it can compete with the combined contributions from the AL and MT terms^3^. With dominating DOS contribution, resistance maximum can appear at higher temperature. Under the application of magnetic field, MT gets suppressed and the resistance maximum is expected to move towards lower temperature with reduced amplitude^6,30^ as observed in the present study.

For zero-field *R*(*T*) measurements while approaching the *T*_c_ from higher temperature, formation of more number of FCPs leads to more reduction in the DOS which may create a pseudogap for the single particle spectrum^49^ and resistance continues to increase. Finally, at certain temperature, a resistance maximum is reached. Further lowering temperature, the superconducting grains in the close proximity combine with each other through Josephson coupling and form superconducting puddles. Here, the contribution from Cooper channel starts to dominate over the single particle channel and resistance continues to drop and at certain characteristic temperature, the superconducting puddles combine altogether and macroscopic phase coherence is established^25,51^. The above situation refers to a single resistive reentrance. However, if the grains are not of uniform dimension all through the sample, there might be multiple reentrances originating from opening and blocking of percolation paths for the localized cooper pairs^25,26^. In this case, for a certain range of temperature, one contribution wins and in the next range of temperature, the other takes over^6,25,27^.

**Figure 5:**
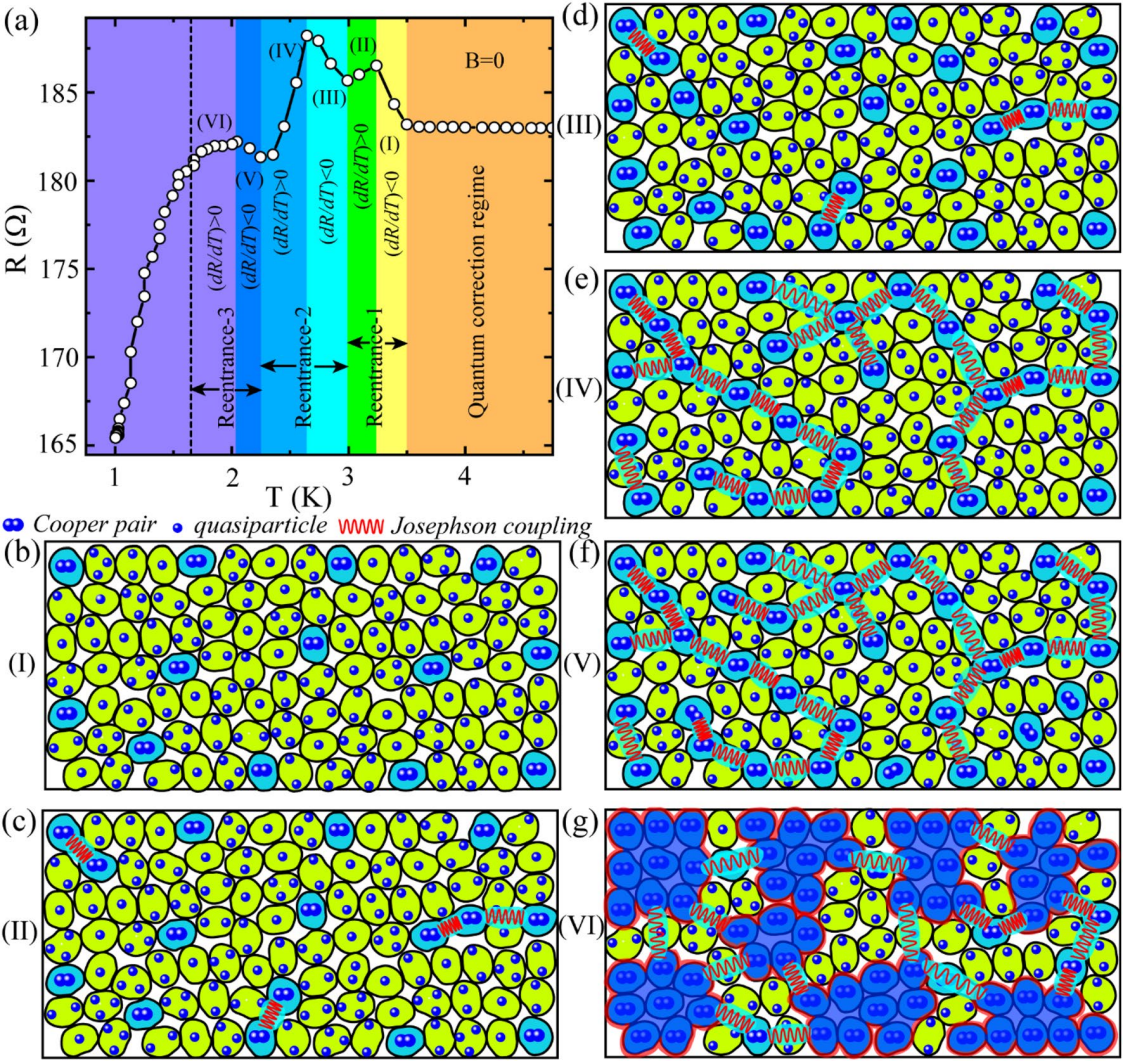
A granularity model has been adopted to explain the reentrant behaviour in  *R*(*T*). (**a**)  *R*(*T*) is fragmented into 6 different regions marked by shaded vertical columns on the basis of sign of  d*R*/d*T* from metallic state (orange shade) to superconducting state (violet colour). Further, these 6 regions are explained by using the granularity model in (**b**)-(**g**), where blue and light green grains are in superconducting (Cooper pairs) and metallic states (quasiparticles), respectively. The red zig-zag lines mark the Josephson coupling between the superconducting grains.

In order to understand the observed reentrant resistive states by using the granular model based on SFs, we have replotted the selective region of the zero-field *R*(*T*) in Fig. 5a with each reentrant regime in two sub-regimes based on an upward (*dR/dT* > *0*) or downward (*dR/dT* < *0*) slopes. Accordingly, the six sub-regimes, marked in ascending order from high to low temperature direction, are explained schematically by the granular model in Fig. 5b–g. As the region-I leads to the first resistance maximum, Fig. 5b suggests the reduction in conduction electrons due to the formation of few scattered FCPs that are confined in the cyan grains. Resistance continues to increase while lowering the temperature as a greater number of electrons get occupied for the FCP formation. With lowering temperature, local superconductivity is established inside these grains and the neighbouring superconducting grains start to couple with each other by Josephson coupling and FCPs start to tunnel from one grain to the other. This leads to a reduced resistance with *dR/dT* > *0* in the region II as shown in Fig. 5c. By further lowering the temperature to region-III, resistance reaches to its maximum value which indicates the rate of formation of FCPs is much more than the rate of coupling between them. The scenario is presented in Fig. 5d which shows a greater number of FCPs but coupling between them is yet to be established. However, by continuously reducing the temperature, localized superconducting grains start to couple with each other and contribute to the conduction and resistance drops in region-IV (Fig. 5e). At this stage, the competition between the Cooper pair channel and the quasiparticle channel becomes almost comparable and with further lowering temperature some more quasiparticles move from single particle channel to form FCPs (Fig. 5f) and in region-V, only a little enhancement in resistance is observed. And finally, in region-VI, the neighbouring superconducting grains couple together to form superconducting puddles of much bigger size. With lowering the temperature, these big superconducting puddles couple with each other by Josephson coupling and the conduction is dominated by the Cooper pairs channel in region-VI as shown in Fig. 5g.

Apart from the appearance of reentrant resistive states in the zero-field *R*(*T*), a resistance peak appears in the *R*(*B*) isotherm around zero field for each of the peak temperatures (*T*_peak_). This leads to a negative MR (*dR/dB* < *0*) region at low field range much lower than the critical field. At the resistance maximum, with the application of magnetic field, the Cooper pairs break into quasiparticles and conductance increases in the single-particle channel. This explains the observed negative MR at the peak temperature *T*_peak_. However, the width of the MR peaks for parallel field is more than that appears for the perpendicular field. This might be due to the stronger detrimental effect for perpendicular field on superconducting pairing and fluctuations than that usually occurs in parallel field^45,52,53^. However, the reason behind the higher amplitude for the negative MR peaks for the parallel field than that occurred for the perpendicular field is not clear and it needs further investigation for clear understanding.

## Conclusion

In conclusion, two-dimensional granular superconducting TiN thin film demonstrates the experimental observation of multiple reentrant resistive states above its transition temperature *T*_c_. The zero-field resistance peaks corresponding to each reentrance lead to low field negative magnetoresistance in the *R*(*B*) isotherms. With the help of low temperature magnetotransport measurements carried out under parallel and perpendicular magnetic field, we show that the origin of the reentrant state lies majorly on the superconducting fluctuations (SFs). The negative MR peaks around zero-field are much more pronounced in the parallel field than that in the perpendicular field direction. Further, it is observed that the widths of these low-field MR peaks remain unaltered with the temperature while their amplitude increases with increasing temperature. We have proposed a granularity-based model on SFs to explain the observed multiple reentrant states that are mainly formed by the competition among different contributions from SFs. The proposed model is able to explain all the observed features and phenomena in the *R*(*T*) and *R*(*B*) measurements except for the enhanced amplitude for the MR peaks with increasing temperature. Further study particularly theoretical modelling would be helpful to understand the evolution of the resistance peaks with temperature and the magnetic field direction.

## Methods

### Thin film growth process

We have used undoped Si (100) substrate covered with Si_3_N_4_ dielectric spacer layer of 80 nm thickness. The Si_3_N_4_ topping layer was grown by using low pressure chemical vapor deposition (LPCVD) technique and in this study, Si_3_N_4_ is the only source of nitrogen for the nitridation of Ti to produce TiN thin films^54–56^. After following up the standard cleaning process of the substrates, Ti films were deposited on the substrate by dc magnetron sputtering using a Ti target of 99.995% purity in the presence of high purity Ar (99.9999%) gas. Sputtering of Ti was performed with a base pressure less than 1.5 × 10^−7^ Torr. Finally, the sputtered samples were transferred in situ to an UHV chamber attached to the sputtering chamber for annealing which was done at ~ 780 °C for 2 h in a high vacuum condition with pressure less than 2 × 10^−7^ Torr. It is noteworthy to mention that the annealing pressure here is much higher than the pressure (< 5 × 10^−8^ Torr) maintained during the annealing for the samples presented in our previous reports^30,55^. Hence, oxygen content in the present batch is expected to be more and it may have significant influence on the transport properties of the sample. The X-ray photoelectron spectroscopy (XPS) analysis carried out on a reference sample (which was placed in the closest proximity of the sample SS2 and grown in the same batch) confirms the presence of oxygen in the sample. The detailed interface studies through XPS characterization are presented in Fig. S7 in the Supporting Information (SI).

### Low temperature transport measurements

For low temperature transport measurements, the thin films were grown into a Hall bar geometry by using the stainless-steel shadow mask. Further, electrical contact leads of Au (80–100 nm)/Ti (5 nm) were deposited by dc magnetron sputtering by using a separate complimentary mask. The dimensions of the sample presented in this study were of 1100 μm length and 500 μm width. The thickness of the film is (6 ± 1) nm which is well below the Ginzburg–Landau coherence length *ξ*_*GL*_(*0*) of about 10 nm as obtained from the upper critical field *B*_*c2*_ and the same is presented in Fig. S6 in the SI.

Low temperature transport measurements were carried out in dilution refrigerator by Oxford Instruments with base temperature 20 mK and equipped with superconducting magnet for magnetic field up to 14 T. For *R*(*T*) measurements, the conventional 4-probe configuration was adopted by using standard Lock-In technique with 100 nA excitation at 17 Hz frequency. Here, model 7265 from Signal Recovery is used as the Lock-in amplifier. Further, a low noise voltage preamplifier (Signal Recovery 5113) was used to record the voltage signal.

### Structural characterizations

For surface morphological characterization, we have used atomic force microscope (AFM) (Multimode V, NS V, Veeco) in tapping mode. X-ray Photoelectron Spectroscopic (XPS) measurements were performed in UHV based Multiprobe Surface Analysis System (OMICRON, Germany) operating at a base pressure of 5 × 10^−11^ Torr. A monochromatic Al-Kα radiation source (1486.7 eV) was employed for data acquisition which was calibrated against C (1s) core level at 284.8 eV. The calibration of work function of the system and the binding energy in photoemission spectra was carried out referring to Au 4f_7/2_ emission line and Au Fermi level. To enhance the sampling depth and minimize the contribution of native oxides and contaminants, in situ sputtering via energetic Ar^+^ ions (at 2 keV) was performed.

### Supplementary Information

Supplementary Information.

## Data Availability

The data that represent the results in this paper and the data that support the findings of this study are available from the corresponding author upon reasonable request.
