# Supplementary material for: Granularity mediated multiple reentrances with negative magnetoresistance in disordered TiN thin films
Source: Sci Rep. 2023 Dec 20;13:22701. doi: 10.1038/s41598-023-50091-7 (PMC10733403; doi:10.1038/s41598-023-50091-7)
Supplement: Supplementary file 1 — Supplementary Information. [file 41598_2023_50091_MOESM1_ESM.pdf]

Supporting Information

Granularity mediated multiple reentrance with negative magnetoresistance in disordered TiN thin films

Sachin Yadav, [1, 2] *R. P. Aloysius,* [1, 2] *Govind Gupta,* [1,2] *and Sangeeta Sahoo**, [1, 2]

[1]*CSIR-National Physical Laboratory, Dr. K. S. Krishnan Marg, New Delhi-110012, India*

[2]*Academy of Scientific and Innovative Research (AcSIR), Ghaziabad- 201002, India*

**Correspondences should be addressed to S. S. (Email: sahoos@nplindia.org)*

Contents:

1. The field dependent *R(T)* measurements under perpendicular field
2. A detailed investigation on the parallel field *R(B)* isotherms
3. Comparison between parallel and perpendicular field magnetoresistance isotherms
4. Magnetic field & temperature dependence of MR(%) obtained from MR isotherms
5. Temperature and magnetic field dependence of the reentrance peaks
6. Calculation of Ginzburg- Landau (GL) coherence length (ξ_{GL}) for TiN sample
7. Interface studies of TiN thin film through X-ray photoelectron spectroscopy (XPS)
8. Surface morphology by atomic force microscopy (AFM) imaging

1. The field dependent *R(T)* measurements under perpendicular field:

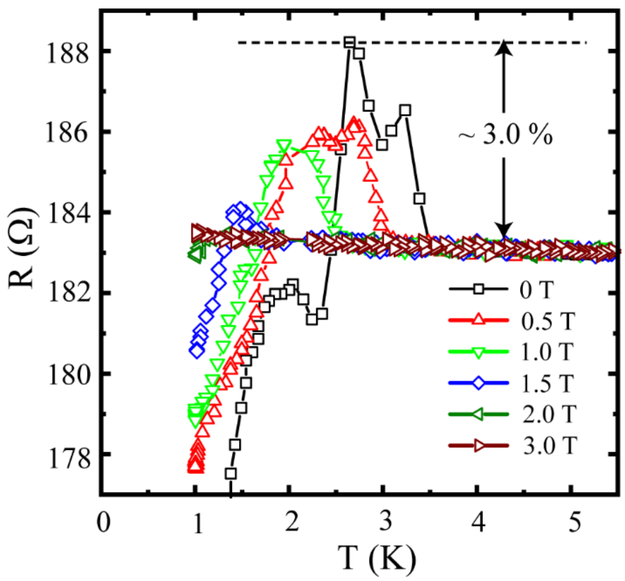

Fig. S1: R(T) data measured under magnetic field applied perpendicular to the sample plane. The maximum resistance difference from the normal state to the highest peak is about ~ 3 % of the normal state resistance (R_N) for the zero-field R(T) and the positions are shown by the dashed line.

An up-turn and corresponding resistance peak in zero-field *R(T)* for disordered materials originate mostly from the quantum interference mediated weak localization (WL) and/or electron-electron interaction (EEI) in the 1D diffusive channel [1-3]. In the presence of perpendicular magnetic field, the aforesaid two quantum phenomena can be distinguished as magnetic field is detrimental for the WL, whereas, EEI remains mostly unaffected/independent of field in the same range where WL gets totally suppressed [3,4]. Hence, the *R(T)* measurements are carried out under magnetic field applied perpendicular to the sample plane and the result is shown in Fig. S1. Here, the evolution of the low-temperature region with magnetic field is highlighted with a particular emphasis on the resistance peaks appeared in the *R(T)*. For zero-field *R(T),* a maximum of about ~ 3% increment in the resistance is observed at the highest resistance peak with respect to the normal state resistance R_N (resistance measured at 5 K). At about 500 mT field, the peaks start to merge together while shifting towards lower temperature with reduced amplitude. With further increasing the magnetic field up to 1T, the resistive peaks get suppressed and at this stage only one

broad peak is visible. At 1.5 T, a very little is seen as per as the peak is concerned but resistance drop due to superconducting fluctuations is clear. With further increase in magnetic field, there is no trace remains for the resistance peak but SF mediated a little drop in the resistance is observed and becomes almost flat under 3 T.

2. A detailed investigation on the parallel field *R(B)* isotherms:

In order to have an insight into the individual feature and assessing its evolution with temperature in more detail, in Fig. S2, we have plotted the same data as that was shown in Fig. 5 but in selective way. In Fig. S2(a), the selected temperature window spans from 1K to 1.9 K and here mainly we emphasize on the progression of the first kink with increasing temperature. As the temperature increases, the positive slope (*dR/dB*) at the lower field, just before the appearance of the first kink, gets suppressed and eventually, no more dip at zero-field is observed at about 1.9 K where the *R(B)* becomes almost flat and it is shown by the black dotted horizontal line. Here, the behaviour is similar to any conventional superconductor where with increasing temperature positive MR due to superconducting fluctuations blends on the normal state at T_c where *R(B)* becomes independent of the applied field.

The flat part, appeared in this lower field region of the *R(B)* at 1.9 K (shown by the green curve), is emphasized in more detail by comparing it with the isotherms measured at temperatures in its close vicinity. In Fig. S2(b), we show four selective *R(B)* isotherms measured at 1.8 K, 1.9 K, 2.0 K and 2.1 K. First at 1.8 K, a small positive MR with a dip near zero-field transforms to an almost flat curve at 1.9 K which is then followed by a negative MR with a peak at zero-field as shown by the red curve measured at 2.0 K. The negative MR continues to appear at 2.1 K too but with reduced amplitude. The other kinks at higher field evolve in a similar way with increasing temperature.

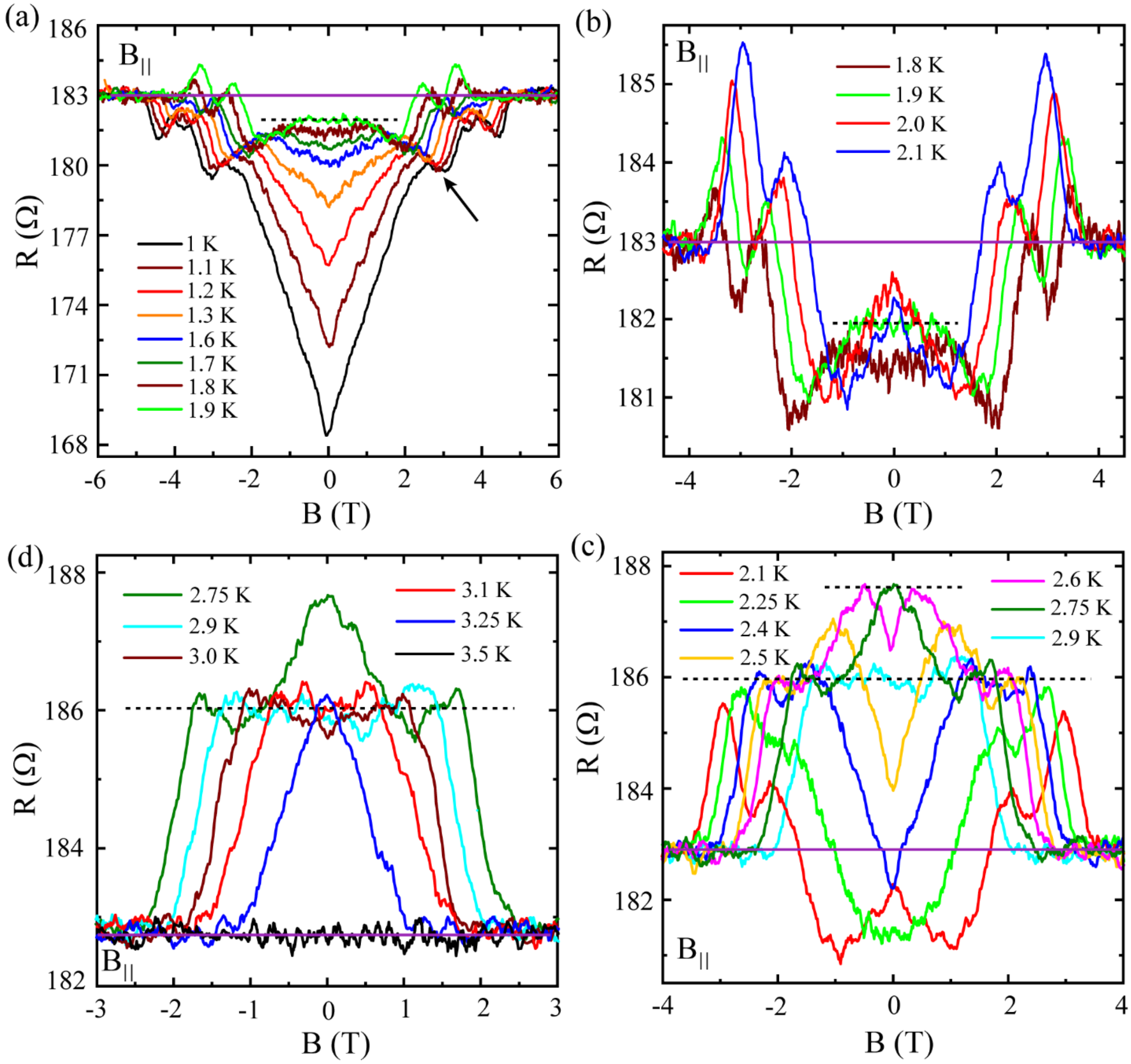

Fig. S2: Different representation of R(B) isotherms presented in Fig. 5 in the main manuscript for parallel magnetic field orientation. The R(B) isotherms are split in four sets with selective temperature window to emphasize the evolution of the specific features individually. These four sets are categorized from low temperature to high temperature in (a)-(d), respectively. The violet solid horizontal line represents the normal state resistance value for the sample. The dotted horizontal lines represent intermediate metastable states. The black arrow shows the evolution of the first kink appearing in the R(B) isotherms while undergoing superconducting to normal state phase transition under the influence of the magnetic field.

Further in Fig. S2(c), we have selected the temperature window of 2.1-2.9 K for the *R(B)* isotherms and we observe that the zero-field negative magnetoresistance peak at 2.1 K becomes almost independent of field in the low field range when measured at 2.25 K, but for relatively higher field, the isotherm follows positive magnetoresistance till it reaches to the next kink. At 2.4 K, the positive magnetoresistance starts from the zero-field and the trace of the first kink disappears. Here, the amplitudes for other higher field kinks become comparable and they fall on the dotted horizontal line corresponding to 186 Ω as already mentioned in Fig. 5 in the main manuscript. At this temperature (2.4 K), the *R(B)* isotherm starts just below the dotted normal state reference line of 183 Ω with a very little portion appearing below it. With further increasing temperature from 2.5 K onwards, the zero-field positive MR gets suppressed and the isotherms go completely above the normal state. At 2.75 K, a negative MR with a peak appearing at zero-field is observed which eventually disappears at 2.9 K and a flat almost field-independent *R(B)* region shows up at low field. This flat part follows the reference line at 186 Ω before it sharply drops to the normal state resistance at higher field which is clearly seen in Fig. S2(d) representing the *R(B)* isotherms for Reentrance-1 regime. With increasing temperature, the span in the field for the flat portion gets reduced and finally it retains only the negative magnetoresistance with a peak at zero field. Here, only the negative magnetoresistance peak appears in *R(B)* before it merges onto the normal state at 3.5 K represented by the violet horizontal line where the *R(B)* isotherm becomes independent of field.

3. Comparison between parallel and perpendicular field magnetoresistance isotherms:

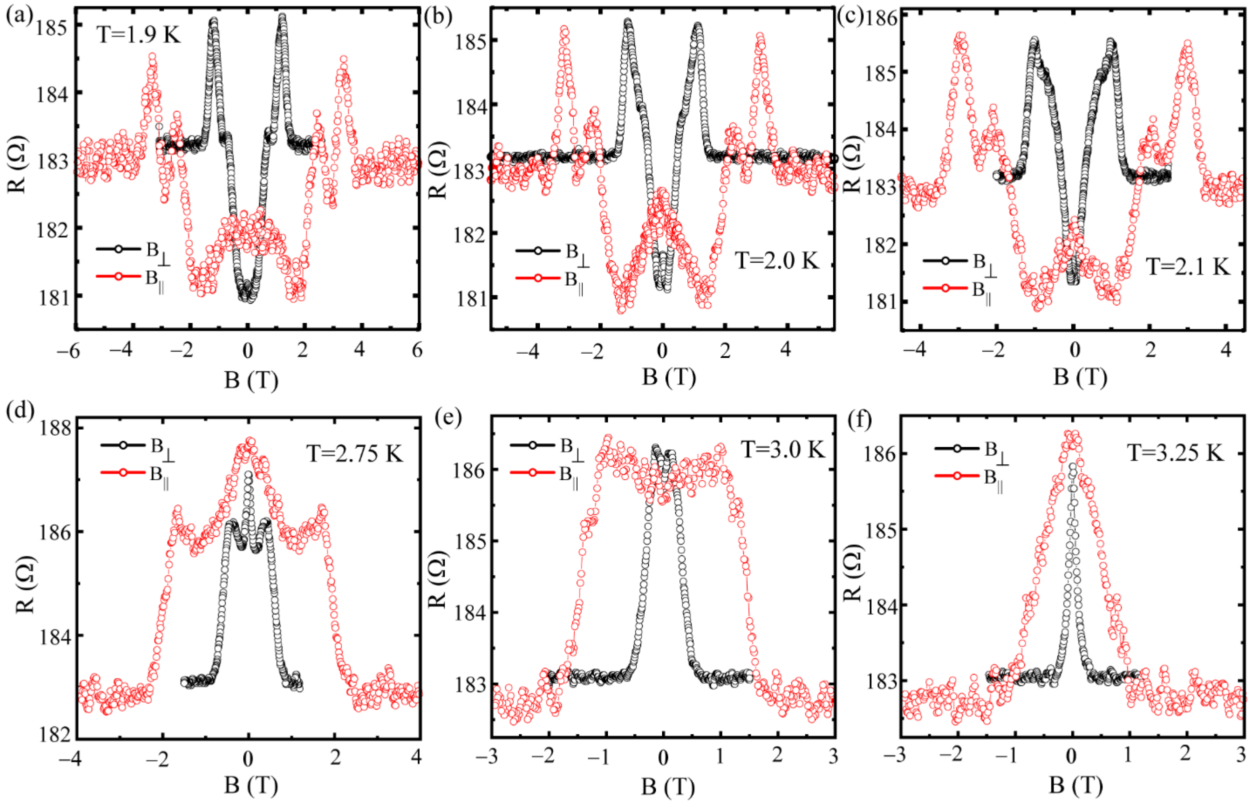

Fig. S3: Comparison of magnetoresistance isotherms measured under perpendicular and parallel magnetic field at same temperature.

4. Magnetic field & temperature dependence of MR(%) obtained from MR isotherms:

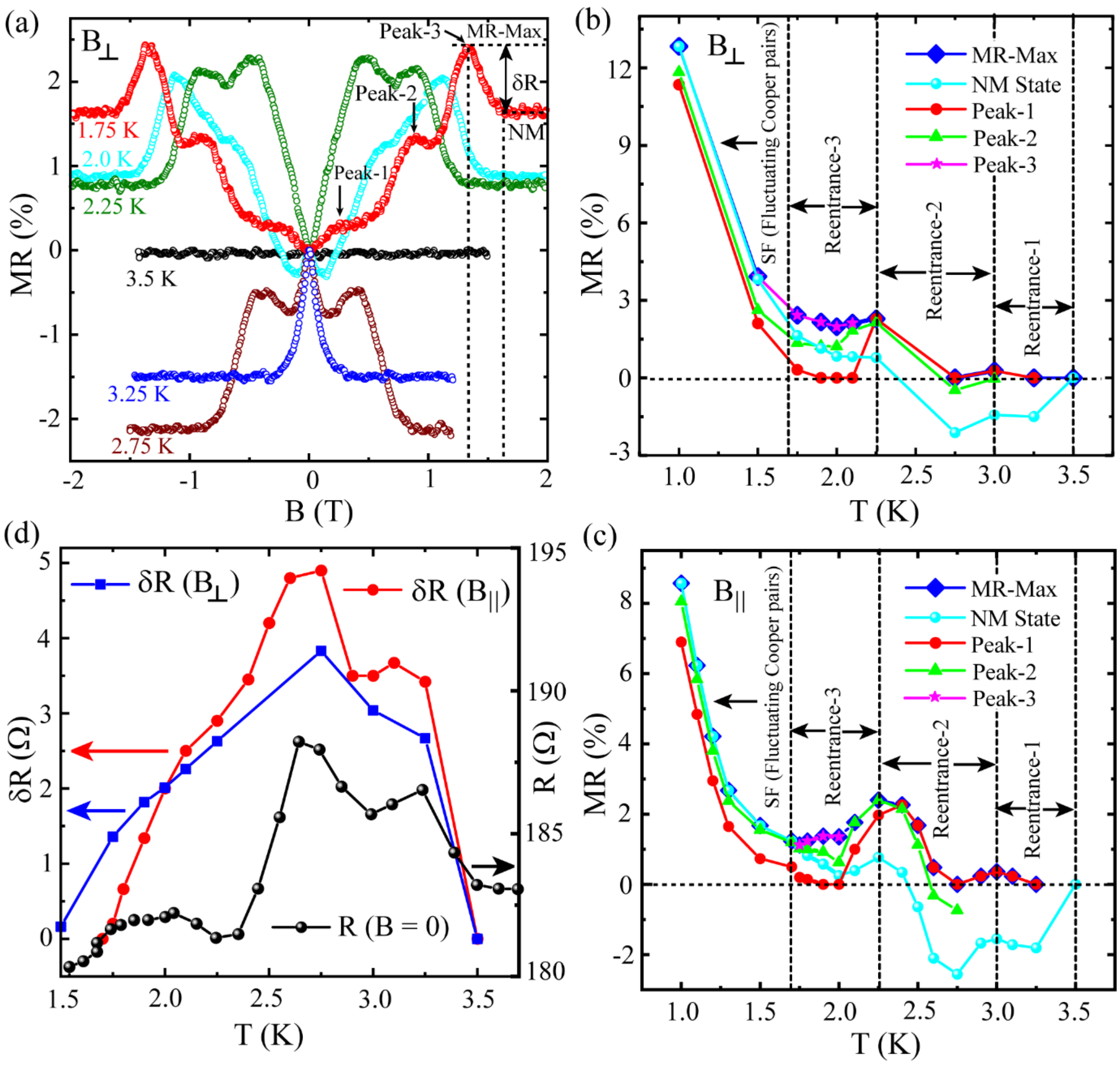

Fig. S4: Magnetic field and temperature evolution of magnetoresistance peaks with a specific emphasis on the percentage change in magnetoresistance $\left[MR(\%) = \left(\frac{R(B)-R(0)}{R(0)}\right) \times 100\right]$ *and its evolution with temperature for perpendicular and parallel field orientations. (a) Field dependence of MR (%) obtained from R(B) isotherms measured under perpendicular field direction for a few representative temperature points. Here, various peak positions, the maximum MR (MR-Max) and the normal state (NM state) positions are defined and marked on the representative curve measured at 1.75 K, as shown by the red open circular points. δR represents the difference in resistance for the MR-Max position from the NM state as shown by the both sided arrows. (b) & (c) Evolution of MR (%) corresponding to all of these defined positions, viz., the MR-Max, NM state, MR peaks etc. with temperature for perpendicular and parallel field orientations, respectively. (d) Comparison of temperature dependences of 'δR' for parallel and perpendicular field with the zero-field R(T).*

We have calculated the magnetoresistance percentage [*MR(%)*] from the measured *R(B)* isotherms by using, $MR(\%) = \frac{R(B)-R(0)}{R(0)} \times 100$, where *R(0)* corresponds to the resistance at zero-field. The field dependence of *MR(%)* for a set of representative isotherms measured under perpendicular magnetic field is shown in Fig. S4 (a). The isotherm measured at 3.5 K represents the normal state where MR becomes independent of field and it is represented by the black curve which acts as the reference level to distinguish positive and negative MR. As there are peaks observed in the *R(B)* isotherms, the field dependent MR also features those peaks. In order to assess the temperature evolution of these peaks in a qualitative way, they are marked and the numbering to the peaks is done from low field to high field direction as shown for a representative isotherm measured at 1.75 K (the red open circular points) in Fig. S4 (a). The MR-Max position, NM state and the difference in the resistance *δR* between these two positions are also shown in Fig. S4 (a) for the same representative isotherm measured at 1.75 K. A zero value of *δR* represents the normal state to have the maximum resistance and a non-zero value of *δR* indicates the presence of a higher resistive state above the normal state. Further, as the peaks evolve with temperature, overall MR evolves around positive and negative values depending on the temperature. There are two extreme cases, for the first one with the minimum resistance at zero-field [i.e., *R(0)* is the minimum resistance for a particular *R(B)* isotherm], the *MR(%)* remains positive for all values of field and for the second one with the maximum resistance at zero-field [i.e., *R(0)* is the maximum resistance for a particular *R(B)* isotherm], the *MR (%)* stays negative for any value of the field. Apart from these two extreme cases, there are isotherms that show both positive and negative values of *MR(%)* at various ranges of fields while measuring the *R(B)* isotherms. Here, we have selectively placed five isotherms in Fig. S4 (a), where two from each of the extreme cases 1.75 K & 2.25 K for the positive value of *MR (%)* and 2.75 K & 3.25 K for the negative value of *MR (%)*) as mentioned above are included and one at 2.0 K represented by the cyan curve shows both type of segments with positive as well as negative values of *MR (%)* in the field dependent MR plot.

The *MR(%)* values at the positions of MR peaks, the MR-Max and the NM state are collected from the field dependent MR isotherms as presented in Fig. S4 (a) and their variations with temperature have been plotted in Fig. S4 (b) & (c), for perpendicular and parallel field orientations, respectively. As defined in Fig. 3(b) in the manuscript, different reentrance regimes with respect to temperature are shown by the vertical dashed lines. Below the Reentrance-3 regime i.e., in the fluctuating Cooper pairs regime, the values for *MR(%)* for all the defined positions appear to be positive and show a strong dependence on temperature. Here, *MR(%)* sharply drops with increasing temperature which happens mainly due to the superconducting fluctuations as the regime is dominated by the fluctuating Cooper pairs. In this regime, the MR-Max and NM state merge as the latter relates to the maximum resistance and here, mostly two very closely spaced peaks (Peak-1 and Peak-2) are present. The third peak starts to show up at the boundary of SF and the Reentrance-3 regimes. Overall, the *MR(%)* at any particular temperature in the SF regime is more for the perpendicular field than that for the parallel field. In the studied range of temperature, *MR(%)* shows an oscillatory type of behaviour with decaying amplitude for higher temperature. From the lower temperature side specifically in the Reentrance-3 regime, three peaks are present, and the second & third peaks merge in Reentrance-2 regime which consists of finally two peaks. Similarly, in the Reentrance-1 regime, all the peaks merge into only one peak, i.e., Peak-1, the closest peak to the zero-field. For both the magnetic field orientations, Peak-1 shows clear oscillatory behaviour with temperature and it touches the horizontal dotted line of zero-MR at the centre of every reentrance regime indicating a resistance peak at zero-field in the measured *R(B)* isotherms. As defined originally, the reentrance regimes were centred around a resistance peak in the zero-field *R(T)* [Fig. 3(b)], here the temperature dependent MR also indicates that the appearance of Peak-1 at zero field occurs at the middle of the reentrance regimes. Hence, the oscillatory behaviour for the Peak-1 consists of a dip in *MR(%)* at the middle of the reentrance regime and a peak in *MR(%)* at the boundary of any two reentrance regimes. Further with increasing temperature, the *MR(%)* related to the normal (NM) state moves down and first it crosses down Peak-3, then Peak-2 and finally it crosses Peak-1. Besides, while moving out of Reentrance-3 regime, *MR(%)* of NM state changes its sign too and it becomes negative and remains negative till it

reaches to the zero-level where the normal state resistance matches with the zero-field resistance. Therefore, with increasing temperature from SF to Reentrance-3, peaks in *R(B)* start to move above the normal state resistance and we obtain the excess resistance 'δR' as defined in Fig. S4 (a).

Finally, we have plotted the temperature variation of 'δR' for perpendicular field (the blue squares) and parallel field (the red circles) in Fig. S4 (d) where we also compare the 'δR' with zero-field *R(T)* (black spheres). We observe that 'δR' varies in a similar fashion for both the field orientations and also, they follow the zero-field *R(T)* characteristic very closely. The peak structures in the zero-field *R(T)* appear also in the temperature variation of 'δR' which is very clear particularly for the parallel field for which more temperature points are available. The appearance of peaks in temperature dependent 'δR' at the same temperature as that appeared in zero-field *R(T)* and also the similar pattern of amplitude modulation of 'δR' & zero-field *R(T)* indicate that the same mechanism is responsible behind the appearance of peak structures in both *R(T)* and *R(B)*. Similar to the MR peak near zero-field, here also 'δR' is more for the parallel field than that for the perpendicular field orientation particularly in the reentrant regions.

5. Temperature and magnetic field dependence of the reentrance peaks:

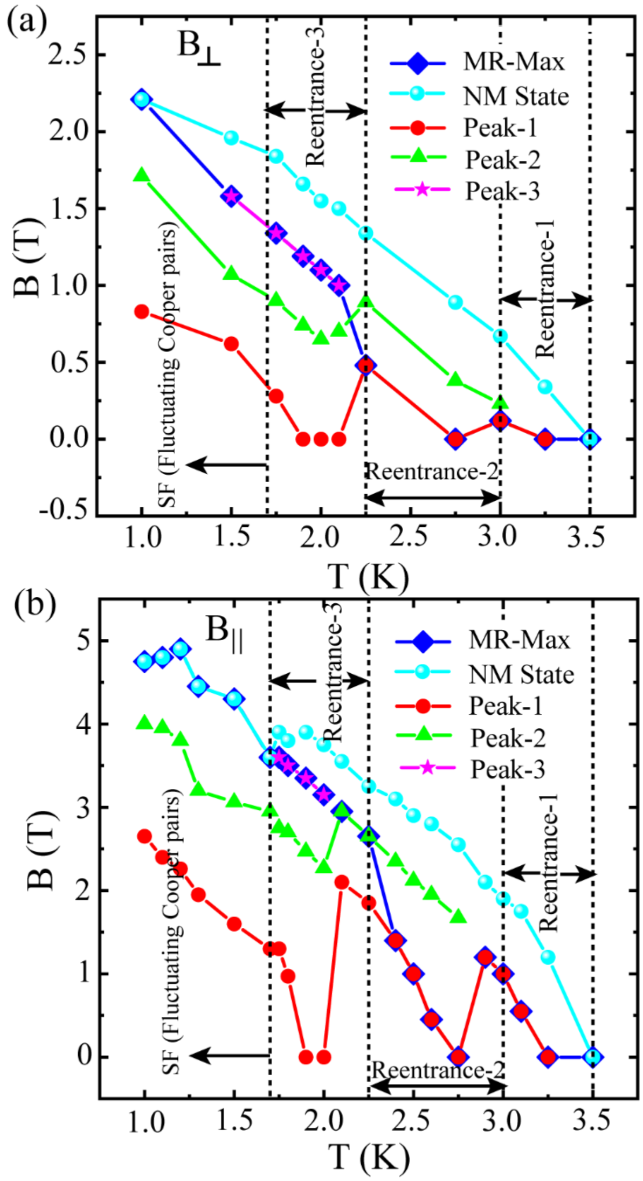

Fig. S5: B-T dependence for the peaks, MR-Max and the NM state for perpendicular field (a) and for parallel field (b).

In addition to the *MR(%)*, we have collected the field values related to the position of MR peaks, MR-Max and the NM state from the *R(B)* isotherms and the corresponding *B-T* dependences are displayed in Fig. S5 (a) & (b) for the perpendicular and parallel field orientations, respectively. First of all, the overall *B-T* dependencies for parallel and perpendicular field show similar trend. However, the characteristic field values for the defined states and the related extent in the field as presented in Fig. S5 for parallel field is almost double of that for perpendicular field which is usual for a conventional anisotropic superconductor where the critical field for parallel field orientation is always higher than the same for perpendicular field orientation [5,6]. As defined, the field value related to an MR peak increases in the order from Peak-1 to Peak-2 to Peak-3 for any particular temperature. Here, the normal state appears always at higher field than the peak positions whereas, the field corresponding to the MR-Max position keeps changing with the temperature. For example, with increasing temperature, first it moves from the NM state to the Peak-3 and then from Peak-3 to Peak-2 and finally to Peak-1 as it is clear in Fig. S5 (a) & (b) for both the field orientations. Further, the region-wise number of MR peaks along with their merging with increasing temperature is clearly evident. For instance, as soon as Peak-3 disappears in Rentrance-3 regime, Peak-2 jumps to follow its path and the same happens in Reentrance-2 regime where Peak-2 disappears and Peak-1 starts to move up in the field position and follows the path from the former. Therefore, with increasing temperature number of MR peaks gets reduced by merging with each other. Further, by merging with higher order (numbered) peaks, Peak-1 displays an oscillatory temperature variation of its field position as obtained from the *R(B)* isotherms.

6. Calculation of Ginzburg- Landau (GL) coherence length (ξ_{GL}) for TiN sample

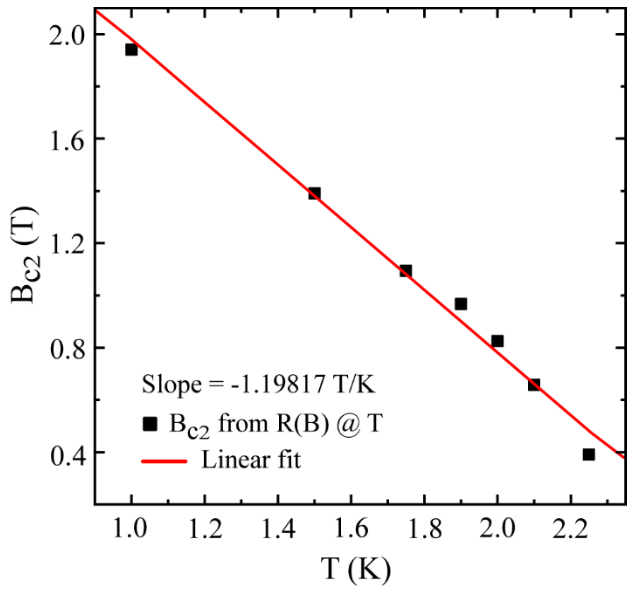

Fig. S6: B-T phase diagram for the TiN sample. Black squares are the data points collected from R(B) isotherms and solid red line represents the linear fit performed on the experimental data points and it provides the slope for calculating the GL coherence length ξ_{GL}.

The Ginzburg-Landau (GL) coherence length $\xi_{GL}(0)$ for the TiN sample (SS2) is calculated by using the standard formula, $\xi_{GL}(0) = \left[\frac{\phi_0}{2\pi T_c \left|\frac{dB_{c2}}{dT}\right|_{T_c}}\right]^{1/2}$, where ϕ_0 is the flux quantum. The experimental data points for the TiN sample are collected from the upper critical field (B_{c2}) values from respective magnetoresistance isotherms. The extracted values from *R(B)* isotherms are fitted linearly in Fig. S6 as shown by the red line. Here, critical temperature (T_c) is taken as 2.75 K from the peak having the maximum resistance in zero-field *R(T)*. The slope obtained from the linear fit has been used for calculating the coherence length $\xi_{GL}(0)$ for the TiN sample and the coherence length is about 10 nm.

7. Interface studies of TiN thin film through X-ray photoelectron spectroscopy (XPS)

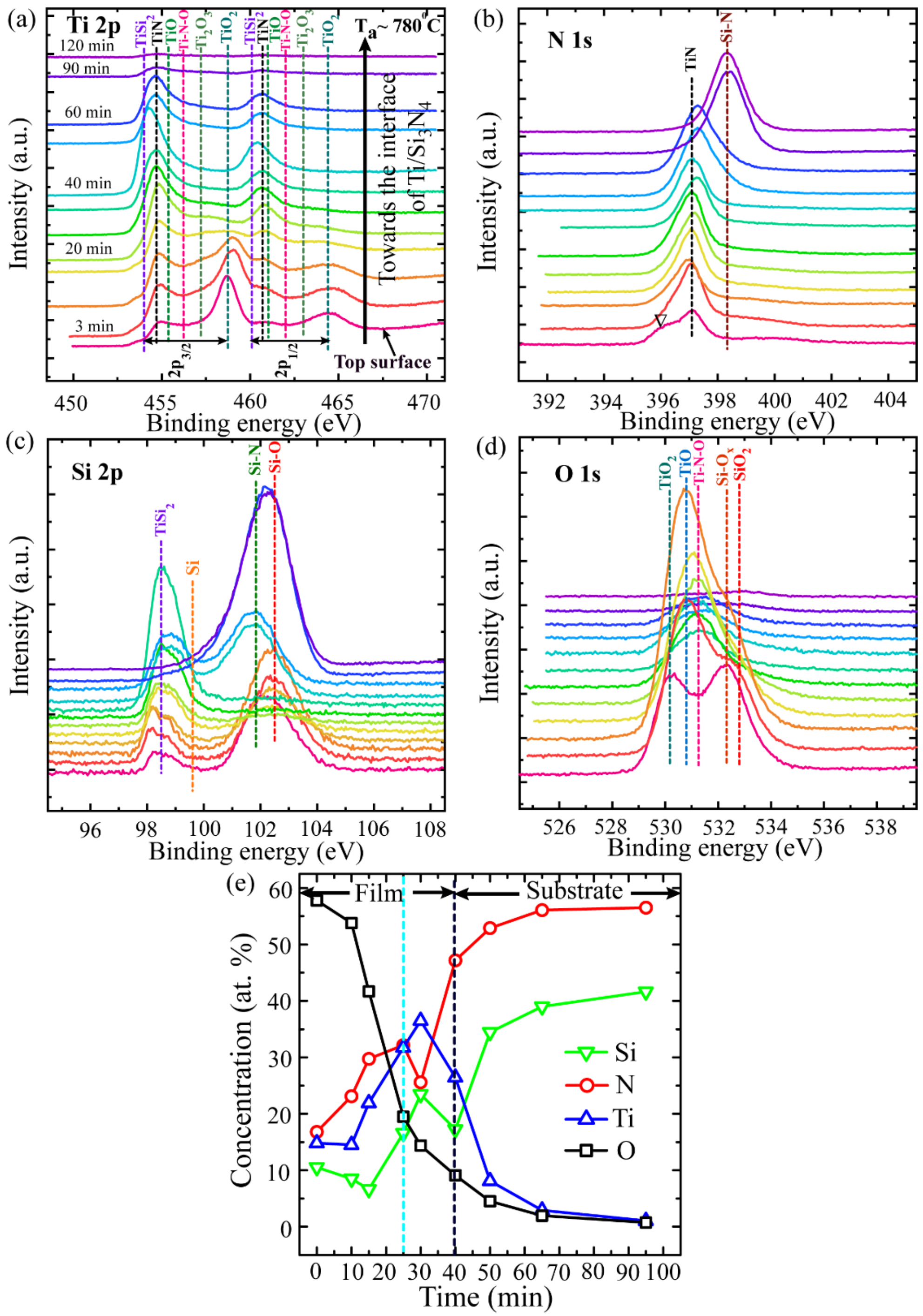

Fig. S7: X-ray photoelectron spectroscopy (XPS) characterization performed on a reference sample which was grown in the same batch with the sample SS2. The core level binding energy spectra of Ti 2p (a), N 1s (b), Si 2p (c) & O 1s (d). The bottom spectrum of each panel refers to the top surface scan for the as-loaded sample and the spectra in the upward direction are measured after etching the film in steps with Ar^{+} ion sputtering. The direction of the XPS scan from the top surface towards the substrate is shown by the black vertical arrow. (e) Variation of atomic percentage for Ti, N, Si and O with respect to etch time. The black dotted vertical line represents the interface while the cyan dotted vertical line refers to the middle of the film.

Here, we present a detailed elemental analysis of a reference sample grown in the same batch by using X-ray photoelectron spectroscopy (XPS) and the corresponding core-level binding energy spectra for Ti 2p, N 1s, Si 2p & O 1s are shown in Fig. S7.

The core level binding energy spectra of Ti 2p are presented in Fig. S7(a), where the scan on the top surface (pink color) for the as-loaded sample shows the dominance of oxide peak at 458.7 eV corresponding to TiO_2[7,8]. With Ar^+ ion etching, the dominance of oxide peak gets reduced, whereas two important peaks show up for $2p_{3/2}$ at binding energy 454.8 eV and for $2p_{1/2}$ at 460.6 eV with a doublet spacing of 5.8 eV which refers to stoichiometric TiN[9,10]. The TiN peaks remain dominant in Ti 2p spectra till the substrate is reached. The binding energy positions for Ti 2p corresponding to its oxidation states (Ti^{2+}, Ti^{3+} & Ti^{4+})[7,8], elemental Ti (Ti^0)[7], $TiSi_2$[8,11] & Ti-N-O[12,13] are marked in Fig. S7(a) along with their doublets. Moreover, the formation of stoichiometric TiN is confirmed by the N 1s spectra as shown in Fig. S7(b), where the initial scan shows the presence of titanium oxynitride (Ti-N-O) at 396.1 eV[12,13] marked with black triangle along with stoichiometric TiN at 397.0 eV[10,12,13]. The TiN peak gets stronger, as we move toward the substrate through the film and the presence of Si_3N_4 substrate becomes evident at 398.0 eV[10,14]. The presence of $TiSi_2$ is confirmed by the Si 2p spectra in Fig. S7(c), where a broad peak consisting of $TiSi_2$ at 98.6 eV[10] & elemental Si at 99.6 eV[15,16] are shown along with silicon nitride (Si-N) at 102.0 eV and silicon oxide (Si-O) at 102.6 eV[10,17]. However, on the top surface scans, the intensity of silicon oxide peak is much more than the peak consisting of silicide & elemental Si. Upon etching across the film, $TiSi_2$ phase becomes more prominent with sharper peak while the intensity of the silicon oxide peak gets significantly reduced. Finally, close to the substrate, the peak related to Si_3N_4 appears at 102.0 eV [17,18] in the Si 2p spectra. The presence of oxide phases in Ti 2p, N 1s & Si 2p spectra is confirmed by the O 1s spectra in Fig. S7(d). Surface scan reveals two prominent peaks corresponding to TiO_2 & $Si\text{-}O_x$ at 530.0 eV[14,19] & 532.2 eV[10], respectively. As, we etch out the top surface, the TiO_2 peak disappears and the peak corresponding to TiO becomes prominent at 530.0 eV[10] along with SiO_2 peak at 532.8 eV[10]. Further, the appearance of Ti-N-O peak inside the film is evident at 531.5 eV[20] and remains prominent till the interface. Here, the dominance of oxygen on the top surface is expected as the sample got exposure to

air while handling for the characterization. However, the oxygen content inside the sample depends mainly on the annealing pressure which was less than 2 x 10^{-7} Torr. We have shown the variation of atomic percentage for Ti, N, Si and O with respect to etch time in Fig. S7(e) where the film-substrate interface is indicated by the black dotted vertical line and the cyan dotted vertical line refers to the mid position of the film. It should be noted that the oxygen content at the middle of the film is about ~ 20% which is much higher than that (< 5%) for the samples annealed with one order better vacuum[10]. Higher oxygen content is shown to suppress the T_c of TiN thin films greatly[21]. Further, the role of oxygen in the resistive reentrance has been also reported for copper oxide based high temperature superconductors[22] which is in support with the observations presented in this article.

8. Surface morphology by atomic force microscopy (AFM) imaging

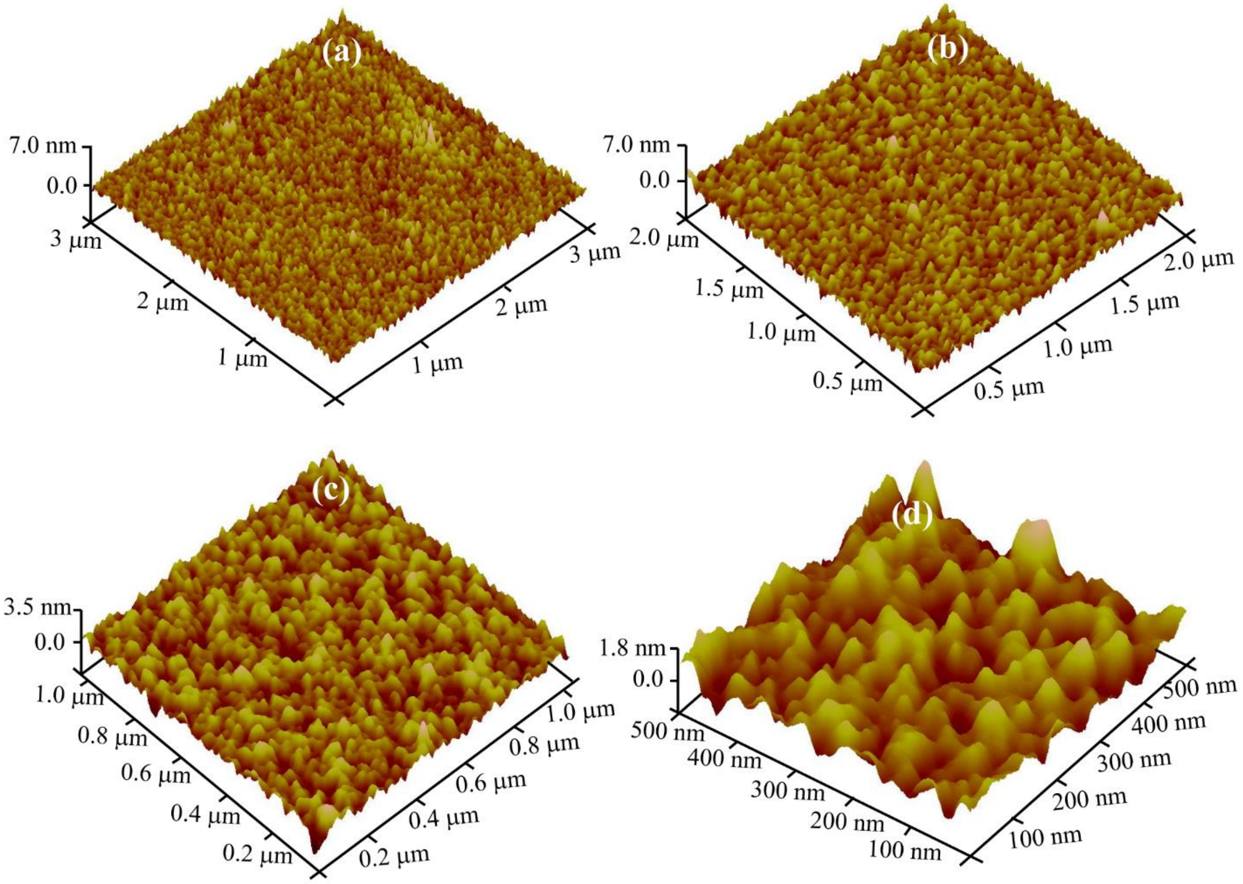

Fig. S8: AFM surface morphology represented in 3D view for the scan area about 3μm × 3μm, 2μm × 2μm, 1μm × 1μm & 500 nm × 500 nm as shown in (a), (b), (c) & (d) respectively.

The surface morphology of the sample SS2 has been studied by atomic force microscopy (AFM) and the same is shown in Fig. S8 for different scan areas of 3µm × 3µm, 2µm × 2µm, 1µm × 1µm and 500nm × 500 nm in (a)-(d), respectively. The granular nature of the sample is evident in the AFM images with average grain size is about $33 \pm 5\ nm$.

References

1 Beloborodov, I. S., Lopatin, A. V., Vinokur, V. M. & Efetov, K. B. Granular electronic systems. *Reviews of Modern Physics* **79**, 469-518, doi:10.1103/RevModPhys.79.469 (2007).

2 Sacépé, B. *et al.* Pseudogap in a thin film of a conventional superconductor. *Nature Communications* **1**, 140, doi:10.1038/ncomms1140 (2010).

3 Yadav, S., Kaushik, V., Saravanan, M. P. & Sahoo, S. Probing electron-electron interaction along with superconducting fluctuations in disordered TiN thin films. *Physical Review B* **107**, 014511, doi:10.1103/PhysRevB.107.014511 (2023).

4 Dauzhenka, T. A., Ksenevich, V. K., Bashmakov, I. A. & Galibert, J. Origin of negative magnetoresistance in polycrystalline SnO${}_{2}$ films. *Physical Review B* **83**, 165309, doi:10.1103/PhysRevB.83.165309 (2011).

5 Joshi, L. M. *et al.* The 2D–3D crossover and anisotropy of upper critical fields in Nb and NbN superconducting thin films. *Physica C: Superconductivity and its Applications* **542**, 12-17, doi:https://doi.org/10.1016/j.physc.2017.08.008 (2017).

6 Farrar, L. S. *et al.* Suppression of superconductivity and enhanced critical field anisotropy in thin flakes of FeSe. *npj Quantum Materials* **5**, 29, doi:10.1038/s41535-020-0227-3 (2020).

7 Mayer, J. T., Diebold, U., Madey, T. E. & Garfunkel, E. Titanium and reduced titania overlayers on titanium dioxide(110). *Journal of Electron Spectroscopy and Related Phenomena* **73**, 1-11, doi:https://doi.org/10.1016/0368-2048(94)02258-5 (1995).

8 Gouttebaron, R. *et al.* XPS study of TiOx thin films prepared by d.c. magnetron sputtering in Ar–O2 gas mixtures. *Surface and Interface Analysis* **30**, 527-530, doi:https://doi.org/10.1002/1096-9918(200008)30:1<527::AID-SIA834>3.0.CO;2-Z (2000).

9 Jaeger, D. & Patscheider, J. A complete and self-consistent evaluation of XPS spectra of TiN. *Journal of Electron Spectroscopy and Related Phenomena* **185**, 523-534, doi:https://doi.org/10.1016/j.elspec.2012.10.011 (2012).

10 Yadav, S. & Sahoo, S. Interface study of thermally driven chemical kinetics involved in Ti/Si3N4 based metal-substrate assembly by X-ray photoelectron spectroscopy. *Applied Surface Science* **541**, 148465, doi:https://doi.org/10.1016/j.apsusc.2020.148465 (2021).

11 Tam, P. L., Cao, Y. & Nyborg, L. XRD and XPS characterisation of transition metal silicide thin films. *Surface Science* **606**, 329-336, doi:https://doi.org/10.1016/j.susc.2011.10.015 (2012).

12 Greczynski, G., Mráz, S., Schneider, J. M. & Hultman, L. Native target chemistry during reactive dc magnetron sputtering studied by ex-situ x-ray photoelectron spectroscopy. *Applied Physics Letters* **111**, 021604, doi:10.1063/1.4993787 (2017).

13 Greczynski, G. & Hultman, L. In-situ observation of self-cleansing phenomena during ultra-high vacuum anneal of transition metal nitride thin films: Prospects for non-destructive photoelectron spectroscopy. *Applied Physics Letters* **109**, 211602, doi:10.1063/1.4968803 (2016).

14 Barshilia, H. C., Ghosh, M., Shashidhara, Ramakrishna, R. & Rajam, K. S. Deposition and characterization of TiAlSiN nanocomposite coatings prepared by reactive pulsed direct current unbalanced magnetron sputtering. *Applied Surface Science* **256**, 6420-6426, doi:https://doi.org/10.1016/j.apsusc.2010.04.028 (2010).

15 Lee, S. M., Ada, E. T., Lee, H., Kulik, J. & Rabalais, J. W. Growth of Ti and TiSi2 films on Si(111) by low energy Ti+ beam deposition. *Surface Science* **453**, 159-170, doi:https://doi.org/10.1016/S0039-6028(00)00339-3 (2000).

16 Poon, M. C., Kok, C. W., Wong, H. & Chan, P. J. Bonding structures of silicon oxynitride prepared by oxidation of Si-rich silicon nitride. *Thin Solid Films* **462-463**, 42-45, doi:https://doi.org/10.1016/j.tsf.2004.05.043 (2004).

17 Cubaynes, F. N. *et al.* Plasma-nitrided silicon-rich oxide as an extension to ultrathin nitrided oxide gate dielectrics. *Applied Physics Letters* **86**, 172903, doi:10.1063/1.1915523 (2005).

18 Zhang, X. & Ptasinska, S. Growth of silicon oxynitride films by atmospheric pressure plasma jet. *Journal of Physics D: Applied Physics* **47**, 145202, doi:10.1088/0022-3727/47/14/145202 (2014).

19 Robinson, K. S. & Sherwood, P. M. A. X-Ray photoelectron spectroscopic studies of the surface of sputter ion plated films. *Surface and Interface Analysis* **6**, 261-266, doi:https://doi.org/10.1002/sia.740060603 (1984).

20 Kuznetsov, M. V., Zhuravlev, J. F. & Gubanov, V. A. XPS analysis of adsorption of oxygen molecules on the surface of Ti and TiNx films in vacuum. *Journal of Electron Spectroscopy and Related Phenomena* **58**, 169-176, doi:https://doi.org/10.1016/0368-2048(92)80016-2 (1992).

21 Torgovkin, A. *et al.* High quality superconducting titanium nitride thin film growth using infrared pulsed laser deposition. *Superconductor Science and Technology* **31**, 055017, doi:10.1088/1361-6668/aab7d6 (2018).

22 Crusellas, M. A., Fontcuberta, J. & Piñol, S. Giant resistive peak close to the superconducting transition in ${\mathit{L}}_{2\mathrm{\ensuremath{-}}\mathit{x}}$${\mathrm{Ce}}_{\mathit{x}}$${\mathrm{CuO}}_{4}$ single crystals. *Physical Review B* **46**, 14089-14094, doi:10.1103/PhysRevB.46.14089 (1992).
